# Supplementary material for: High-Performance Photodetectors Based on the 2D SiAs/SnS2 Heterojunction
Source: Nanomaterials (Basel). 2022 Jan 24;12(3):371. doi: 10.3390/nano12030371 (PMC8840698; doi:10.3390/nano12030371)
Supplement: Supplementary file 1 [file nanomaterials-12-00371-s001.zip › nanomaterials-1529511-supplementary.pdf]

nanomaterials

Supporting Information

High-Performance Photodetectors Based on the 2D SiAs/SnS2 Heterojunction

Yinchang Sun [1], Liming Xie [2,3], Zhao Ma [1], Ziyue Qian [2,3], Junyi Liao [2,3], Sabir Hussain [2], Hongjun Liu [1], Hailong Qiu [1,*], Juanxia Wu [2,*] and Zhanggui Hu [1,*]

1 Tianjin Key Laboratory of Functional Crystal Materials, Institute of Functional Crystal, Tianjin University of Technology, Tianjin 300384, China; acencore@163.com (Y.S.); 18837161430@163.com (Z.M.); hjliu@email.tjut.edu.cn (H.L.)
2 CAS Key Laboratory of Standardization and Measurement for Nanotechnology, CAS Center for Excellence in Nanoscience, National Center for Nanoscience and Technology, Beijing 100190, China; xielm@nanoctr.cn (L.X.); qianzy2019@nanoctr.cn (Z.Q.); liaojy2018@nanoctr.cn (J.L.); sabirphys@yahoo.com (S.H.)
3 University of Chinese Academy of Sciences, Beijing 100049, China
* Correspondence: qiu@tjut.edu.cn (H.Q.), wujuanxia@nanoctr.cn (J.W.); hu@tjut.edu.cn (Z.H.)

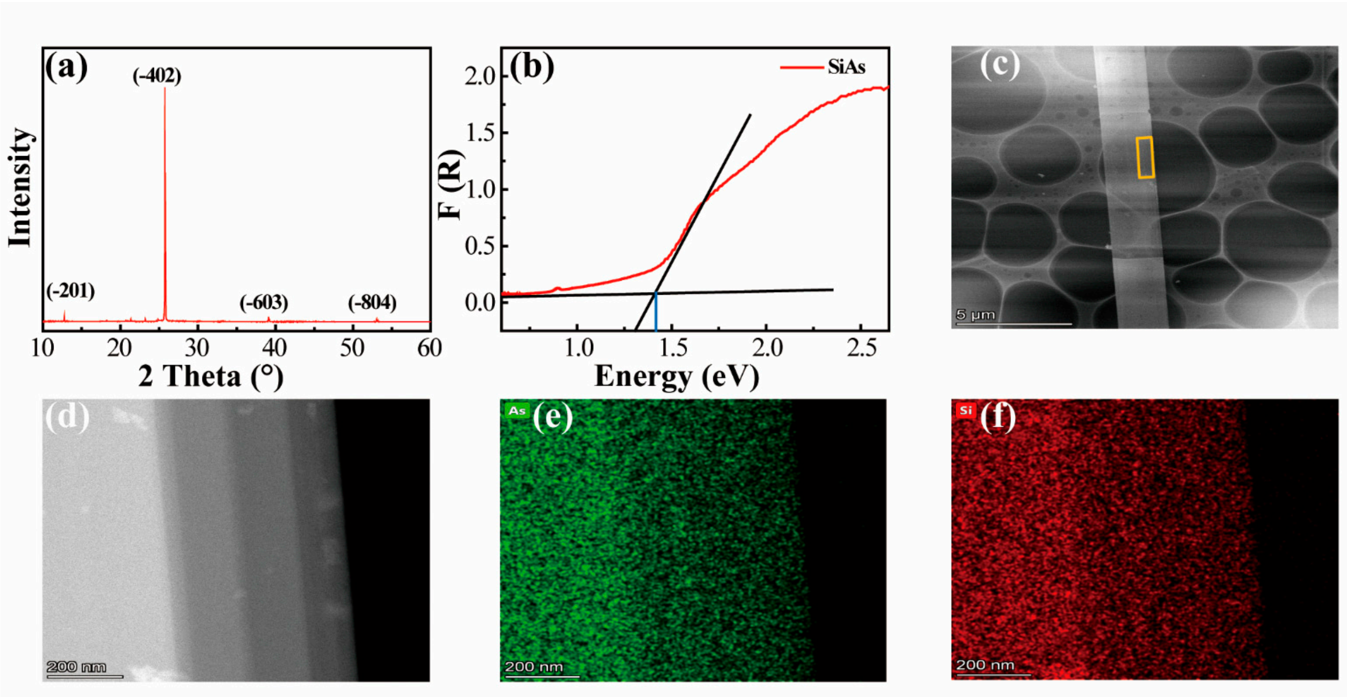

Figure S1. (**a**) X-ray diffraction (XRD) pattern of strip SiAs crystal. (**b**) The ultraviolet diffuse reflectance spectrum of SiAs polycrystalline powder. (c) Low-magnification image of striped SiAs crystal. (**d**) The picture of the significant part in figure (**c**) shows the delamination phenomenon. The upper right illustration shows the EDX element mapping between Si atoms and As elements. The lower illustration shows that Si:As is close to 1:1. (**e**–**f**) EDX element mapping of As (**e**) and Si (**f**) atoms.

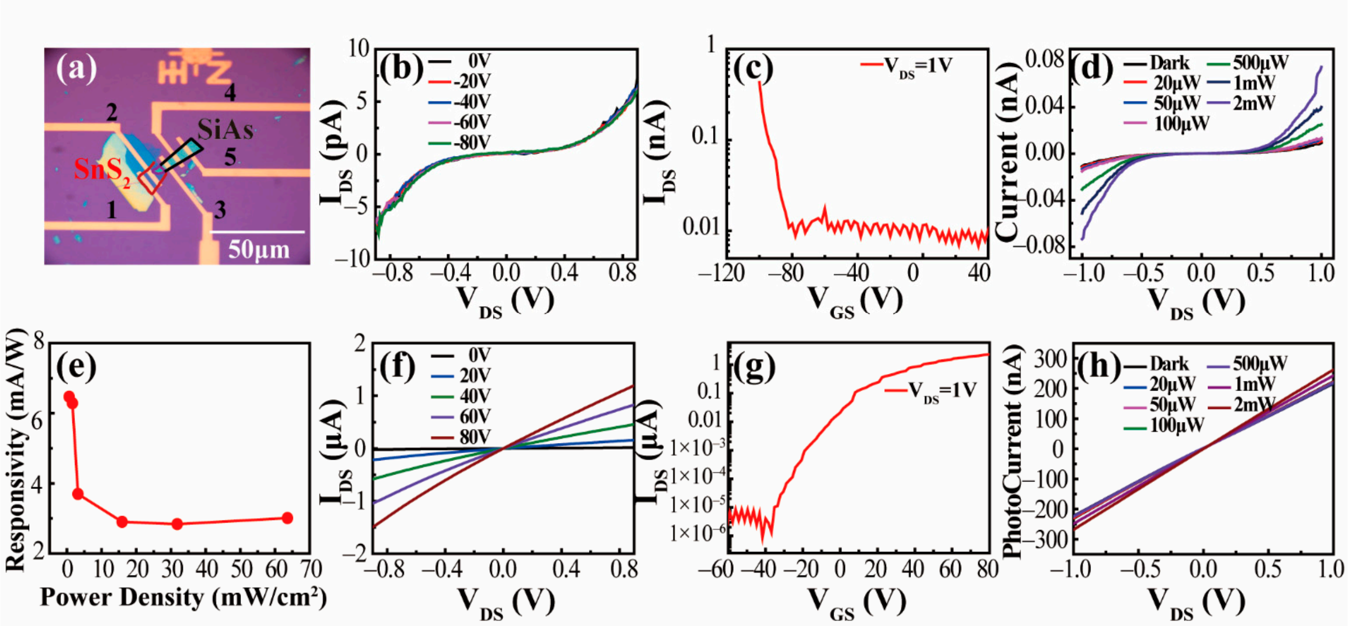

Figure S2. (**a**) SiAs-SnS_2 heterojunction morphology picture. (**b**) The output curves of p-SiAs crystal under different gate voltages. (**c**) When V_{DS}= 1V, the transfer characteristic curve of SiAs device. (**d**) When V_{GS}=0V, the relationship between photocurrent and V_{DS} of 4/5 electrode pairs (SiAs devices) under different incident light powers. (**e**) The photoelectric responsivity of SiAs devices. (**f**) The output curves of the SnS_2 device under different gate voltages. (**g**) Transfer characteristic curve of n-SnS_2 when V_{DS} = 1V. (**h**) The relationship between photocurrent and V_{DS} of n-SnS_2 under different incident optical power.

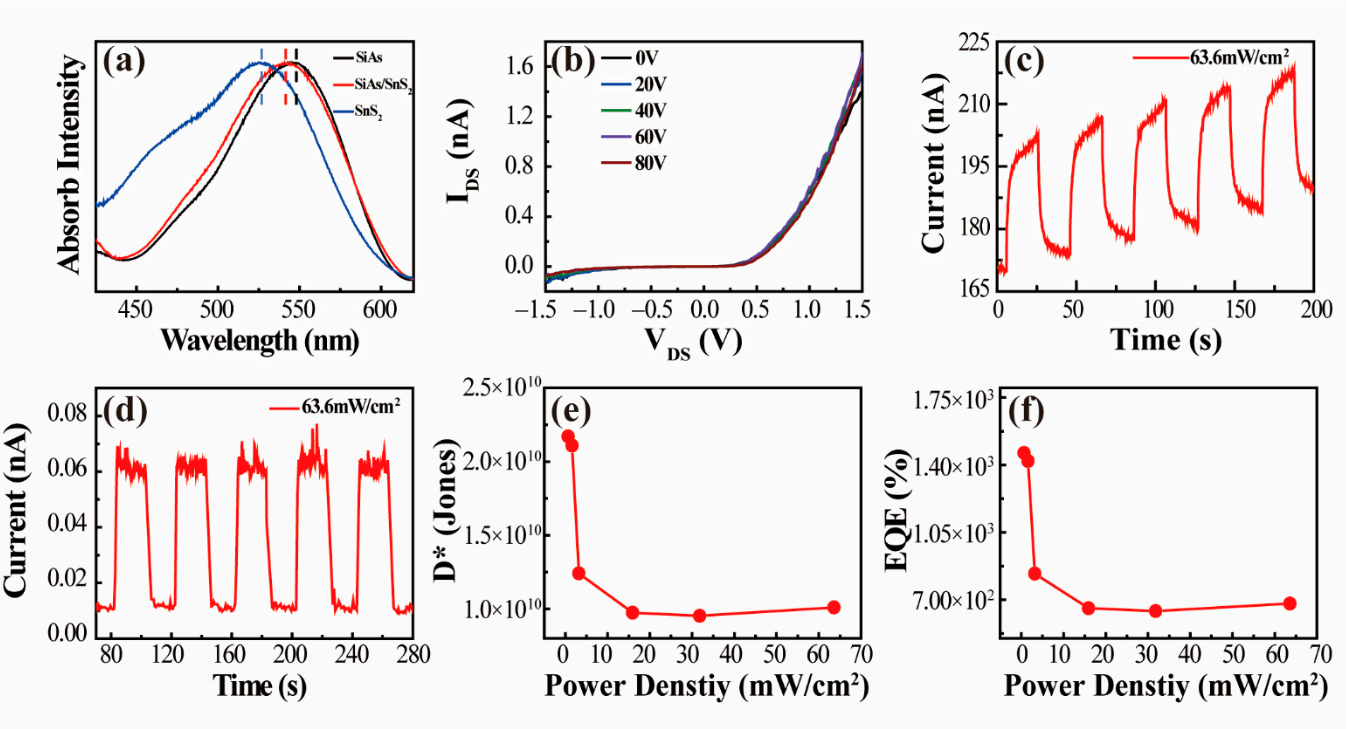

Figure S3. (**a**) Absorbance spectra of different structural regions after normalization. (**b**) Output characteristic curve of 2 / 4 electrodes pair for comparison. (**c**–**d**) Time resolution curves of SnS_2 devices and SiAs devices. (**e**–**f**) The relationship between *D** and *EQE* of SiAs device under 550nm laser irradiation and incident optical power density.
